# Wnt Signaling Activates Shh Signaling in Early Postnatal Intervertebral Discs, and Re-Activates Shh Signaling in Old Discs in the Mouse

**DOI:** 10.1371/journal.pone.0098444

**Published:** 2014-06-03

**Authors:** Tamara Winkler, Eric J. Mahoney, Debora Sinner, Christopher C. Wylie, Chitra Lekha Dahia

**Affiliations:** 1 Tissue Engineering Regeneration and Repair Program, Hospital for Special Surgery, New York, New York, United States of America; 2 Emeritus Professor, Division of Developmental Biology, Cincinnati Children's Hospital Medical Center, Cincinnati, Ohio, United States of America; 3 Division of Orthopaedic Surgery, Cincinnati Children's Hospital Medical Center, Cincinnati, Ohio, United States of America; 4 The Perinatal Institute Division of Neonatology, Perinatal and Pulmonary Biology, Cincinnati Children's Hospital Medical Center, Cincinnati, Ohio, United States of America; Leibniz Institute for Age Research - Fritz Lipmann Institute (FLI), Germany

## Abstract

Intervertebral discs (IVDs) are strong fibrocartilaginous joints that connect adjacent vertebrae of the spine. As discs age they become prone to failure, with neurological consequences that are often severe. Surgical repair of discs treats the result of the disease, which affects as many as one in seven people, rather than its cause. An ideal solution would be to repair degenerating discs using the mechanisms of their normal differentiation. However, these mechanisms are poorly understood. Using the mouse as a model, we previously showed that Shh signaling produced by nucleus pulposus cells activates the expression of differentiation markers, and cell proliferation, in the postnatal IVD. In the present study, we show that canonical Wnt signaling is required for the expression of Shh signaling targets in the IVD. We also show that Shh and canonical Wnt signaling pathways are down-regulated in adult IVDs. Furthermore, this down-regulation is reversible, since re-activation of the Wnt or Shh pathways in older discs can re-activate molecular markers of the IVD that are lost with age. These data suggest that biological treatments targeting Wnt and Shh signaling pathways may be feasible as a therapeutic for degenerative disc disease.

## Introduction

Intervertebral discs (IVD) consist of a multilayered fibrocartilaginous annulus fibrosis (AF) surrounding a semi-liquid nucleus pulposus (NP), in which cells derived from the embryonic notochord [1], [2] are surrounded by a proteoglycan-rich, water-retaining matrix (reviewed in [3]). During aging, structural defects arise in the AF [4], which lead to herniation of the NP, usually into the spinal canal. This most often occurs in the lower lumbar region, where the herniated NP compresses the cauda equina, a complex of spinal nerves supplying body segments below L1 (reviewed in [5]). In addition, loss of the NP causes the herniated disc to compress, which crushes the spinal nerves emerging between the two vertebrae that are normally kept apart by the disc. The neurological consequences vary, but include lower back pain, and loss of sensation and motor function of structures innervated by the affected spinal nerves (reviewed in [6]). It is estimated that one in seven people suffer from the symptoms of degenerative disc disease (DDD).

The most effective treatment of DDD would be to stimulate regeneration of the disc using the developmental mechanisms that previously controlled its normal growth and differentiation. However, these mechanisms are not well understood. We have used the murine disc to identify signaling pathways active during disc growth and differentiation, and have shown previously that the NP cells synthesize many signaling ligands during postnatal growth and differentiation [7]. In particular, SHH, secreted by the NP cells, is essential for cell proliferation in the growing disc and for the synthesis of its differentiation markers, including the transcription factors Brachyury (BRA) and SOX9 as well as the extracellular matrix components of the disc [8]. During this study we also found that blockade of the Shh pathway led to a corresponding increase in canonical Wnt signaling in the disc, as shown by the TOPGal reporter mice. These data raise the questions of what controls Shh expression and/or function in the disc, and how these controls change with age, leading to age-related changes in the disc.

Here we show, both *in vitro* and *in vivo*, that canonical Wnt signaling is an essential activator of Shh signaling in the disc. Furthermore, we show that both pathways become down-regulated with age, concomitant with a decline in expression levels of extracellular matrix and other differentiation markers in the disc. To test whether the age-related down-regulation was permanent, we tested the effect of activating the canonical Wnt pathway in older discs, at one year of age. This had the effect of re-activating Shh signaling and expression of its downstream targets. These data suggest that biological therapies for age-related changes in the IVD of humans may be possible in the future.

## Results

### Wnt signaling in the NP regulates Shh signaling and its targets

**Figure 1:**
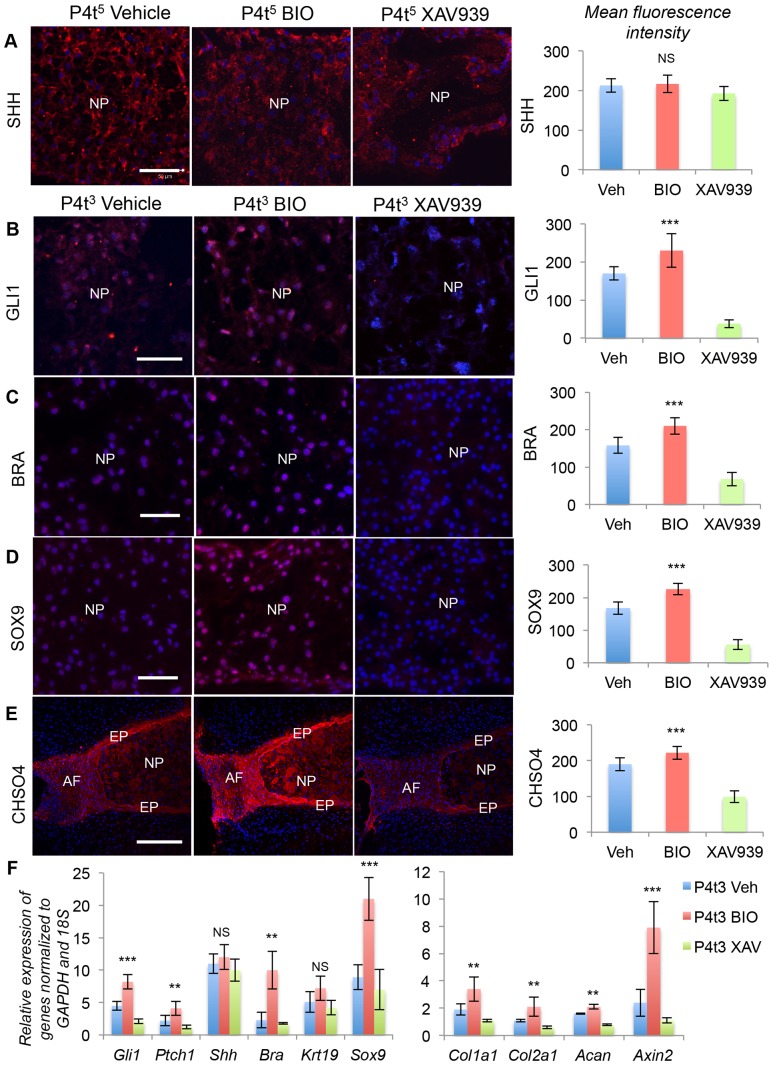
Wnt signaling regulates Shh signaling and its targets in vitro. A–E show protein expression levels of SHH (A), GLI1 (B), BRA (C), SOX9 (D), and CHSO4 (E) after the treatments shown at the top of each column. Postnatal day four (P4) IVDs were cultured for either five days (P4t^5^) or three days (P4t^3^), in either vehicle alone, BIO, or XAV939. The panels on the right of each row show quantitation of pixel intensity measurements from three independent experiments, each carried out in duplicate. F shows qPCR data from the same experiments. Scale bar in A-D = 50 µm, E = 200 µm. Nuclei are stained blue with POPO3-iodide. NP  =  nucleus pulposus, AF  =  annulus fibrosus, EP  =  end plate. (* p≤0.05, ** p≤0.01, *** p≤0.001, NS =  not significant).

We have showed that blockade of Shh signaling resulted in up-regulation of canonical Wnt signaling in postnatal day four (P4) mouse IVDs [8]. To test the hypothesis that these two pathways functionally interact during postnatal growth and differentiation of the IVD we tested the effects of manipulation of Wnt signaling both in vitro and in vivo. First, using an organ culture system established in our lab [8] we tested *in vitro* the effects of the small molecules BIO and XAV939 on IVDs [8]. BIO activates canonical Wnt signaling by inhibiting GSK3β and hence stabilizing β-catenin [9], [10], whereas XAV939 inhibits canonical Wnt signaling by activating Axin2 [11], resulting in degradation of β-catenin. We established the doses required to regulate Wnt signaling in the NP cells in vitro (data not shown). BIO (30 µM) and XAV939 (400 µM) were used, since these doses altered expression levels of activated β-catenin and AXIN2 (downstream target of canonical Wnt signaling) in NP and AF cells of P4 cultured IVDs. Using these conditions, we cultured P4 IVDs for three or five days (P4t^3^ or P4t^5^) either in vehicle only, 30 µM BIO, or 400 µM XAV939. The results are shown in Fig. 1A–E, first as sample immunofluorescence images and second as quantitative differences based on pixel intensity measurements of the fluor (see Materials and Methods for details). Neither treatment caused an obvious difference in the expression of SHH protein, as shown by immunofluorescence staining (Fig. 1A). Next, we assayed the expression of downstream targets of Shh signaling. Expression of GLI1, BRA and SOX9 proteins were dramatically reduced following blockade of canonical Wnt signaling by XAV939 (Figs. 1B–D). IVDs treated with BIO had higher expression levels of these markers compared to vehicle only controls. Next we assayed levels of chondroitin sulfate (CHSO4) proteoglycan by immunostaining (Fig. 1E). Blockade of canonical Wnt signaling resulted in reduced levels of CHSO4 in the P4 IVD. The expression of CHSO4 is much higher in the NP and AF, and hence with quantifiable laser setting was not seen in the lumbar vertebral growth plate chondrocytes (LV-GP), but is shown as a separate image Figure. S1. RNA was isolated from NP cells dissected from the cultured IVDs from different treatment groups, and subjected to qPCR. The results are shown in Fig. 1F. Expression of the Wnt target Axin2 was dramatically increased by BIO, and reduced by XAV939 treatment, showing that the agonist and antagonist were both acting as expected. Expression of Gli1 and Ptch1, both of which are signaling components in the Shh pathway, as well its downstream targets, was increased by BIO, but reduced by XAV939 treatment. Expression of other targets of Shh such as Col1a1 and Col2a1, Bra and Sox9, was similarly up-regulated by BIO and down-regulated by XAV939. Expression of aggrecan (Acan) was also reduced. In contrast no significant difference in the mRNA levels of Shh and Cytokeratin19 (Krt19) were observed following manipulation of Wnt signaling, suggesting that either canonical Wnt signaling regulates Shh signaling downstream of the ligand, or somehow maintains its local concentration. These experiments do not discriminate between these two possibilities.

**Figure 2:**
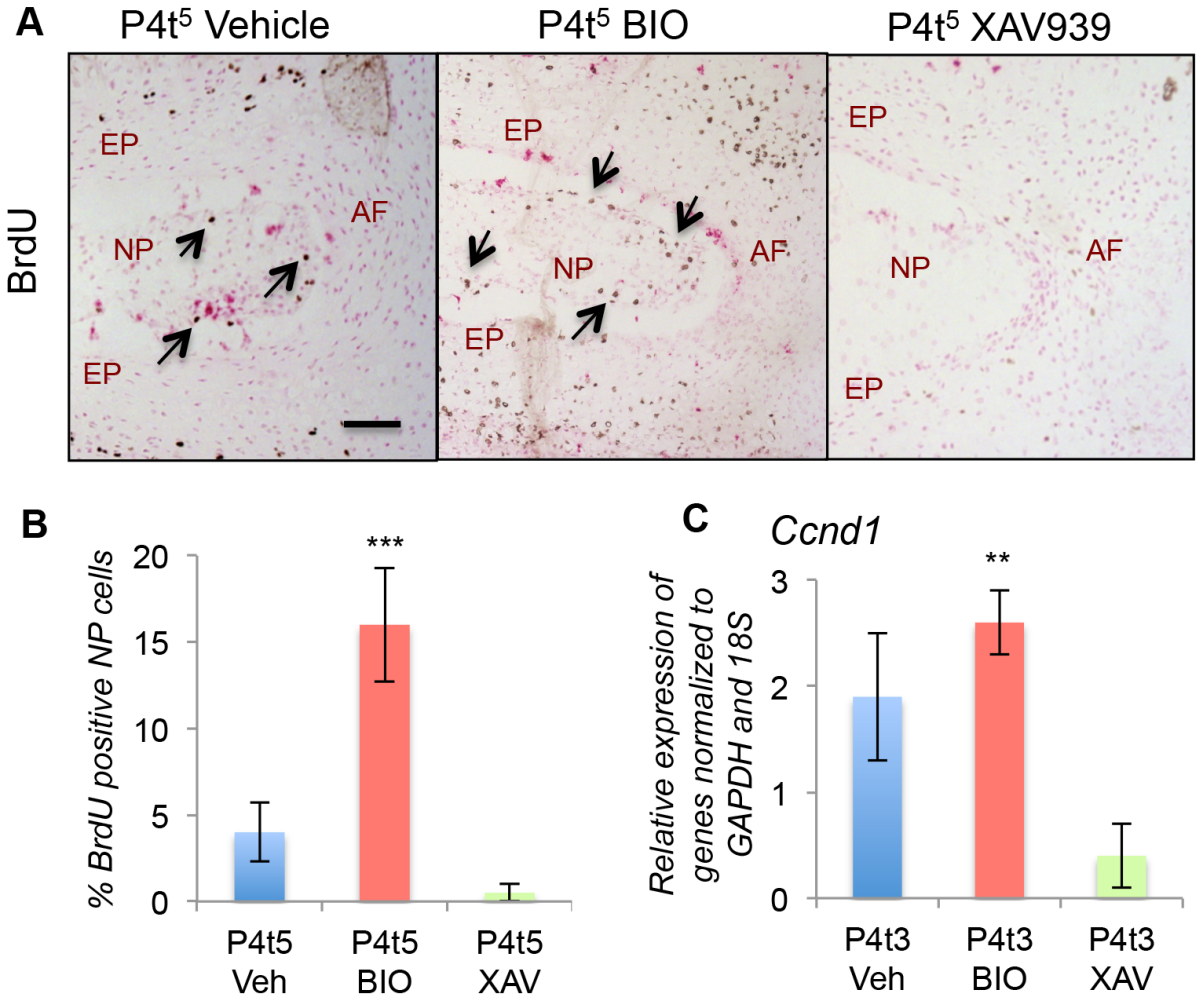
Wnt signaling regulates NP cell proliferation. A shows BrdU incorporation (brown nuclei) by P4 IVDs cultured in vehicle only, BIO, or XAV939. Nuclei are counterstained with nuclear fast red. B shows the percentages of nuclei containing BrdU, counted in serial sections from three independent experiments, each carried out in duplicate. C shows expression levels of cyclin D1, assayed by qPCR, from the same experiments. NP  =  nucleus pulposus, AF  =  annulus fibrosus, EP  =  end plate. (** p≤0.01, *** p≤0.001).

We have demonstrated that Shh signaling is both necessary and sufficient for cell proliferation in the NP during postnatal growth [8]. Therefore we assayed for cell proliferation following activation or blockade of canonical Wnt signaling. IVDs were pulsed with BrdU 24 hrs before snap freezing and cryosectioning. BrdU immunostaining showed an increase in the numbers of proliferating cells (brown nuclei) after activation of Wnt signaling, and complete loss of proliferating cells after blockade of canonical Wnt signaling (Fig. 2A & B). Increase in BrdU positive cells following treatment with BIO was observed in the other components of the IVD; the end plate and, inner layer of AF, as well as the growth plate. If Wnt regulates hedgehog, then increase in cell proliferation in the Shh targets would be expected. In addition, it is well established that hedgehog signaling regulates cell proliferation in the growth plate (reviewed in [12], [13]) hence addition of BIO results in increased BrdU incorporation by growth plate chondrocytes too. Quantification of the data is shown in Figure 2B. qPCR analysis of Cyclin D1 (Ccnd1) mRNA expression in the NP also showed similar results. Ccnd1 mRNA expression was significantly (p≤0.01) reduced in IVDs treated with XAV939 compared to vehicle only BIO treated discs (Fig. 2C). These data show that blockade of canonical Wnt signaling, which results in loss of Shh signaling and its targets, also results in the loss of cell proliferation the NP cells.

**Figure 3:**
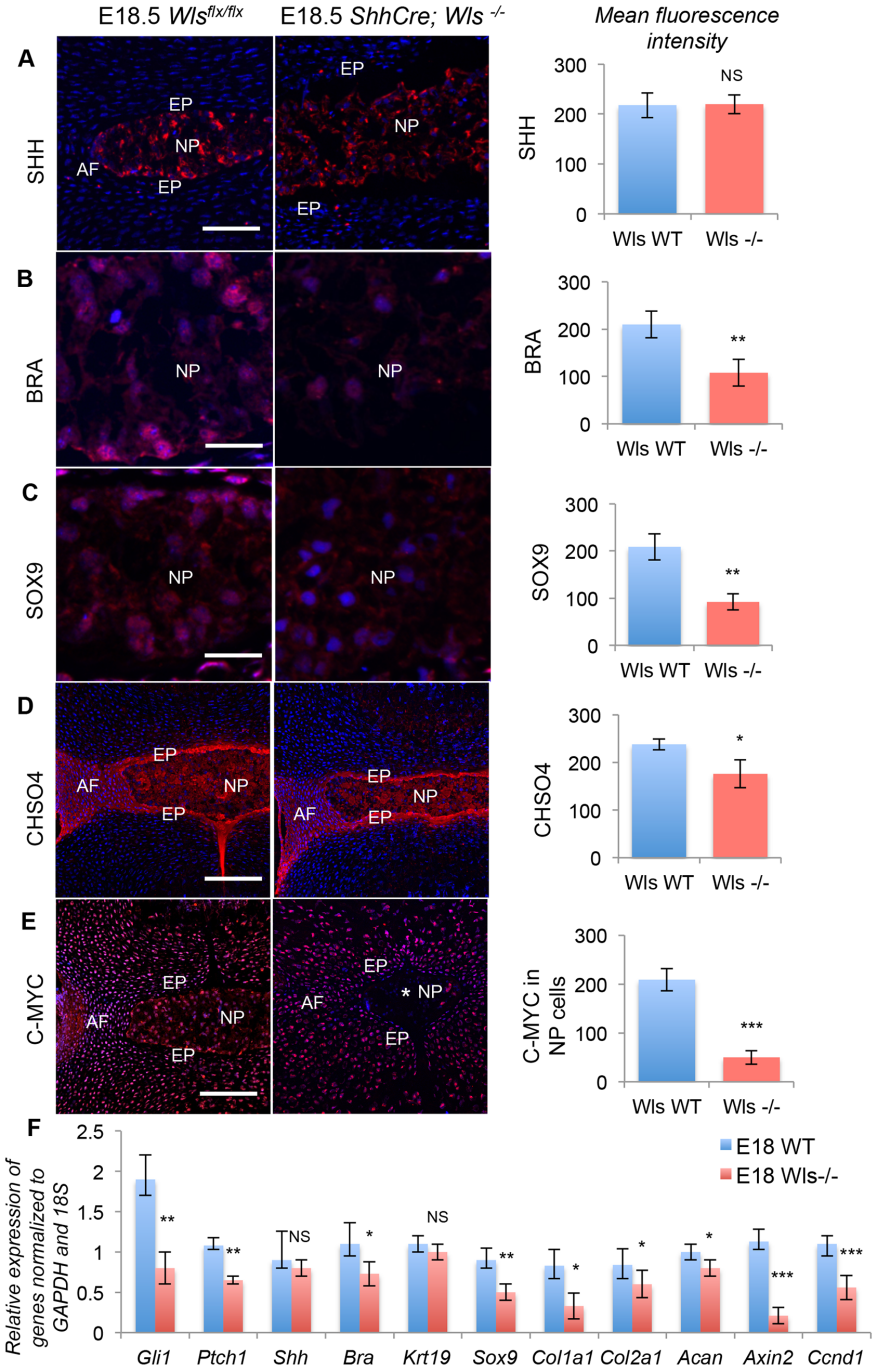
Wnt signaling regulates Shh signaling and its targets in vivo. A–E show expression levels, assayed by immunofluorescence, of SHH (A), BRA (B), SOX9 (C), CHSO4 (D) and C-MYC (E), in E18.5 IVDs from either *Wls^flx/flx^* (control, left hand column) or *Wls-/-* (conditional knock-out, center column) embryos. The right hand column shows quantification of mean fluorescent intensities of the IVD markers of *Wls^flx/flx^* (blue bars) and *Wls* ^−*/*−^ (red bars) IVDs from three independent experiments. F shows qPCR quantification of mRNA levels of IVD markers from *Wls^flx/flx^* (blue bars) and *Wls* ^−*/*−^ (red bars) IVDs from the same experiments. Scale bar in A, D & E = 200 µm, B & C = 20 µm. Nuclei are stained blue with POPO3-iodide. NP  =  nucleus pulposus, AF  =  annulus fibrosus, EP  =  end plate. (* p≤0.05, ** p≤0.01, *** p≤0.001, NS =  not significant).

In vivo targeting of Wnt signaling was accomplished using the ShhCre; Wls ^flx/flx^ mice [14]. WLS (Wntless) protein is required for the secretion of WNT proteins from the cells synthesizing them [15]. Shh driven Wls-null pups die soon after birth due to severe respiratory stress. Therefore, IVDs were collected at E18.5. The IVDs had fully formed and segmented in both the wild-type (Wls^flx/flx)^ and the Wls mutants (Wls^−/−^) at this stage. Immunofluorescence staining showed no change in the expression of SHH in the Wls^−/−^ NPs (Fig. 3A). However, expression of Shh signaling targets was drastically reduced in the Wls^−/−^ NPs, including BRA (Fig. 3B), SOX9 (Fig. 3C) and CHSO4 (Fig. 3D). C-MYC, a known Wnt target was observed to be lost only in the NP cells (Fig. 3E) of the Wls^−/−^ IVDs confirming that the Wnt signaling was specifically targeted in the NP cells. qPCR analysis of NP cells dissected from lumbar IVDs of both the wild-type and mutant mice confirmed the reduction in Shh signaling and its targets (Fig. 3F). Gli1, Ptch1, Bra, and Sox9 were all reduced at the mRNA level. qPCR analysis also showed a decrease in the expression of Col1a1, Col2a1, Acan, Axin2, and Ccnd1. In contrast and similar to the *in vitro* studies no significant change in the expression of Shh or Krt19 in the Wls mutant NP cells was seen.

Taken together, the results from both the in vitro and in vivo experiments show that Shh signaling in the NP during postnatal growth and development of the IVD is controlled by Wnt signals produced by the NP.

### Shh and Wnt signaling are both down-regulated with age

**Figure 4:**
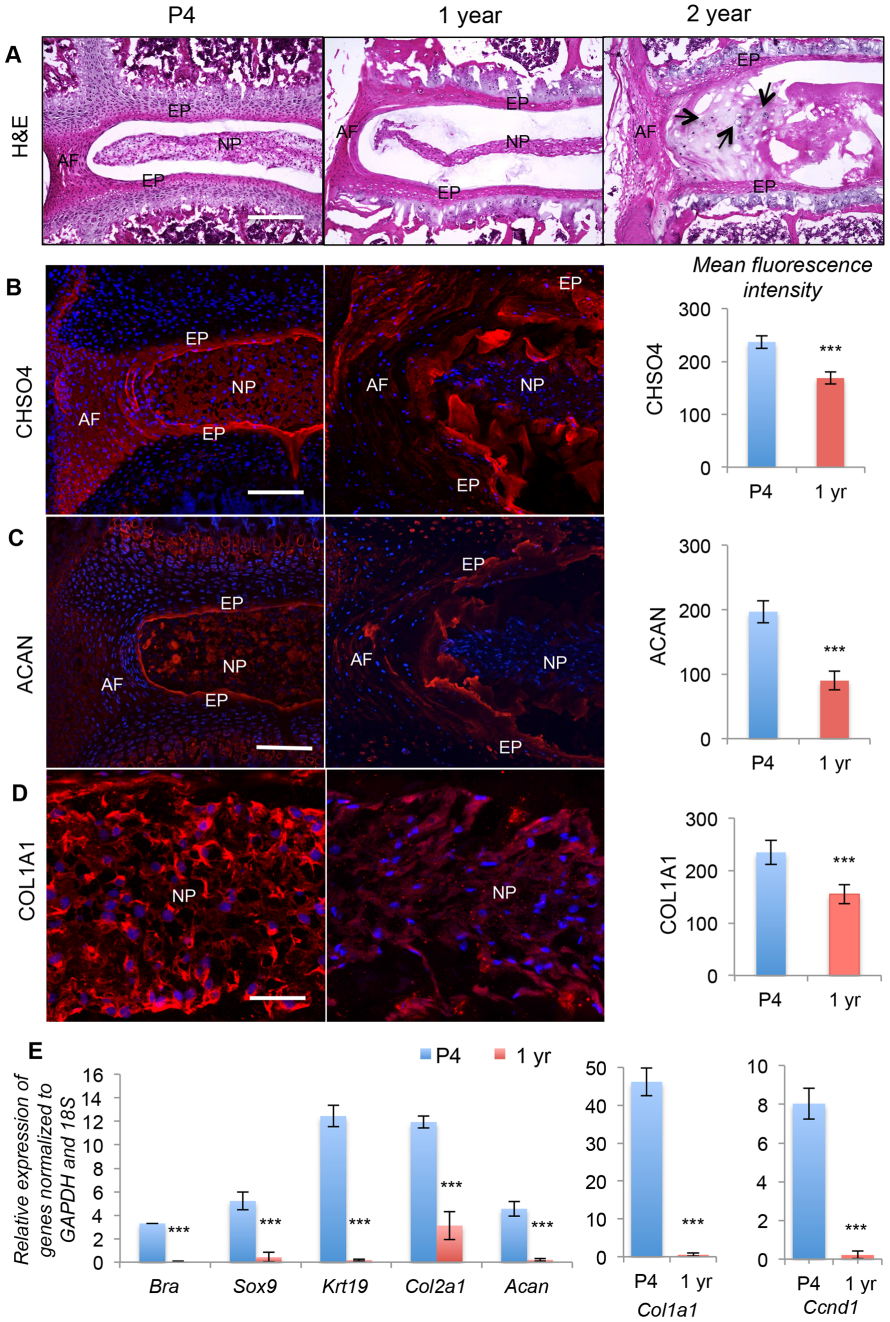
Age related changes in IVD differentiation markers. A shows the normal histological structure of the IVD at four days (P4), one year, and two years of age. B–D show the distribution (by immunofluorescence) and quantities (by fluorescent intensity measurements from three independent experiments, each carried out in duplicate) of CHSO4 (B), aggrecan (ACAN, C) and collagen 1 (COl1A1, D) at P4 and one year. E shows mRNA levels, assayed by qPCR of IVD markers at P4 (blue bars) and one year of age (red bars). Data are from three independent experiments, each carried out in triplicate. In A, B and C scale bar = 200 µm, and in D scale bar = 50 µm. Nuclei are stained blue with POPO3-iodide. NP  =  nucleus pulposus, AF  =  annulus fibrosus, EP  =  end plate. (* p≤0.05, ** p≤0.01, *** p≤0.001, NS =  not significant).

We next tested the hypothesis that cessation of growth and differentiation results from down-regulation of the Wnt and Shh pathways. Rapid growth of the IVD ends approximately nine-weeks after birth [2]. By one year of age, many changes in histological structure and marker expression are found. These include a dramatic compaction of the NP cells into a central mass in the disc space (Fig. 4A), surrounded by a glycosaminoglycan (GAG)-rich matrix (shown for chondroitin sulfate in Fig 4B). By two years of age the NP cells could no longer be identified, and the IVD was filled with chondrocyte-like cells, and loose matrix (right hand panel in Fig. 4A). Many markers identified as downstream targets of Shh signaling during the rapid growth period have reduced levels of expression by one year of age. These were assayed by immunostaining (shown for CHSO4 in Fig. 4B, ACAN in Fig. 4C, COL1A1 in Fig. 4D), or by qPCR (shown for Bra, Sox9, Krt19, Col2a1, Acan, Col1a1, and the Wnt target (and cell cycle marker) Ccnd1 (Fig. 4E).

**Figure 5:**
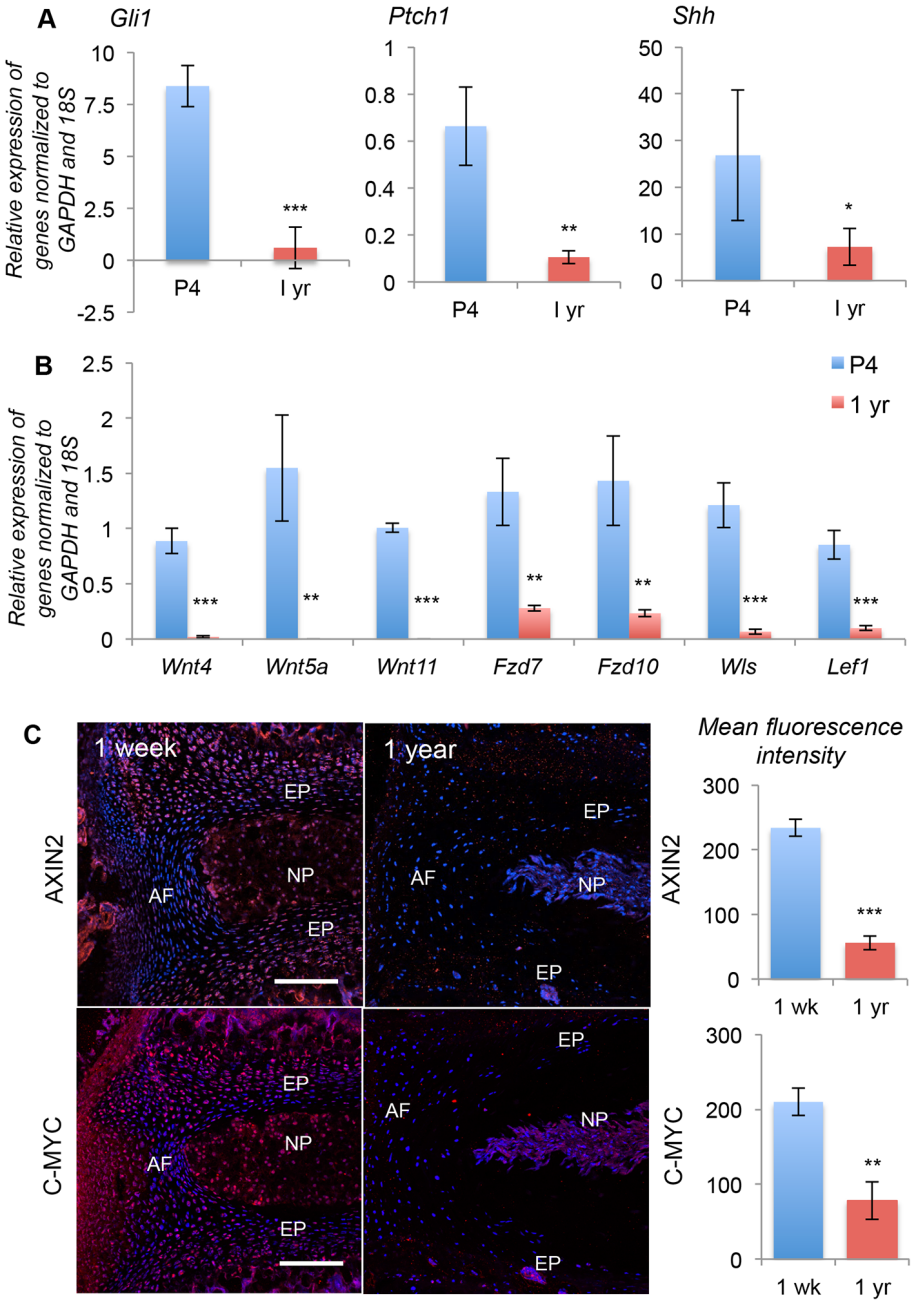
Wnt and Shh signaling are both lost with age. A shows mRNA levels, assayed by qPCR, of *Shh* and its targets at P4 and one year of age. B shows mRNA levels of *Wnt* ligands, receptors, and targets in NP cells at P4 and one year of age. C shows proteins levels, by immunostaining, of Wnt targets AXIN2 and C-MYC, at one-week and one year of age. Fluorescence intensities from three independent experiments, each in triplicate, are quantitated in the right hand panels. In C scale bar  =  200 µm, and nuclei are stained blue with POPO3-iodide. NP  =  nucleus pulposus, AF  =  annulus fibrosus, EP  =  end plate. (* p≤0.05, ** p≤0.01, *** p≤0.001).

We next tested the levels of Wnt and Shh signaling at one year of age, compared to their levels during rapid growth (P4). Figure 5 shows that the components of Shh and Wnt signaling are highly active during early postnatal growth and differentiation and are down-regulated by one year of age. qPCR analysis (Fig. 5A and B) showed a reduction in expression of Gli1 and Ptch1 between the period of rapid growth (P4) and post-growth stages (one year of age). Expression of Shh itself was also reduced (Fig. 5C). We also analyzed the expression levels of components of Wnt signaling pathways by qPCR. Figure 1B shows down-regulation of Wnt4, Wnt5a, Wnt11, in one-year-old mouse NP cells compared to P4. Wnts −2, −2b, and −7 were also analyzed, but they were not expressed in the NP cells at either of the ages analyzed (data not shown). Expression of the Wnt receptors Fzd7 and Fzd10 was also reduced at one year compared to from P4, in the NP cells. qPCR analysis for Fzd1 did not detect a signal at either P4 or one year (data not shown). The expression of Wls was also reduced with age, as were the Wnt targets Lef1, AXIN2 (Fig. 5B and C) and C-MYC (Fig. 5C). These data show that both Wnt and Shh signaling are down-regulated by one year of age, as are their targets, in the NP of the mouse IVD.

### Activation of Wnt signaling at one year of age reactivates Shh signaling and its targets

**Figure 6:**
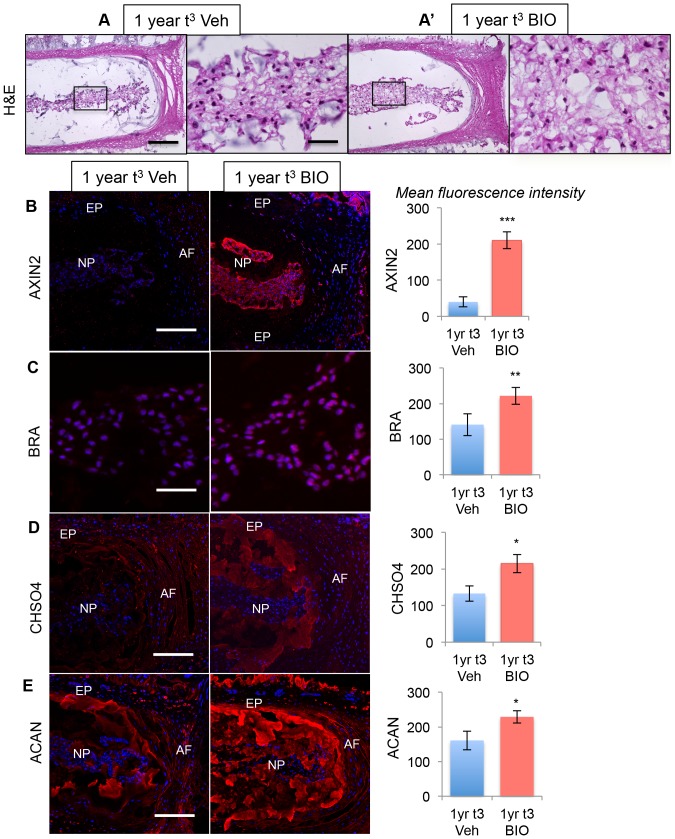
Activation of Wnt signaling in older IVDs re-activates Shh signaling and its targets. A shows the changes in histological structure of IVD at lower magnification and NP cells shown in inset at higher magnification (A and A′), and levels of AXIN2 (B), BRA (C), CHSO4 (D) and ACAN (E) after treatment of one-year-old IVDs with BIO. Right hand panels show quantitation of fluorescent intensities in control (blue bars) and BIO-treated (red bars) NP cells. The data represent three independent experiments, each carried out in duplicate. Scale bar in A and C  =  50 µm, and B, D and E  =  200 µm. In B - E nuclei are stained blue with POPO3-iodide. NP  =  nucleus pulposus, AF  =  annulus fibrosus, EP  =  end plate. (* p≤0.05, ** p≤0.01, *** p≤0.001).

**Figure 7:**
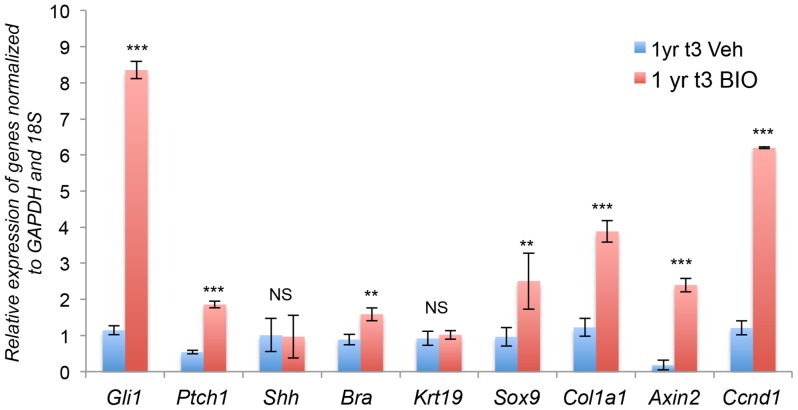
Activation of Wnt signaling in older IVDs re-activates Shh signaling and its targets at transcriptome level. The figure shows mRNA levels, assayed by qPCR from three independent experiments, each carried out in duplicate, of *Shh* and its targets in NP cells from one-year-old mice, cultured for three days (1yr t3) either in the presence of vehicle (blue bars) or BIO (red bars). (* p≤0.05, ** p≤0.01, *** p≤0.001, NS =  not significant).

Biological therapy for disc degeneration, using the same signaling pathways that control normal growth and differentiation, is a very attractive proposition. However, this would require the response pathways to developmental signals to remain present in post-growth period discs. To test this hypothesis, we artificially activated Wnt signaling in one-year-old discs in vitro, and assayed the response with respect to histological appearance, activity of the Shh pathway, and expression of Shh targets. Lumbar IVDs from one-year-old mice were cultured in the presence of vehicle or 30 µM BIO for three days (1yr t^3^). The IVDs were washed and either snap frozen for cryosectioning, or the NP cells were dissected and processed for RNA isolation. H&E staining of mid-coronal sections through the IVD shows that, following activation of canonical Wnt signaling, the NP cells started to lose their highly compacted structure and regained the reticular structure seen during growth, whereas IVDs treated with vehicle only were unchanged (Fig. 6A and A′). Up-regulation of Wnt signaling by BIO was confirmed by increased expression of AXIN2, which was significantly (p 0.001) increased following treatment with BIO (Fig. 6B). Immunofluorescence showed an up-regulation in expression of BRA (Fig. 6C), CHSO4 (Fig. 6D), and ACAN (Fig. 6E). qPCR data showed that expression of the Shh targets Gli1 and Ptch1 were both up-regulated (Fig. 7), demonstrating that increased canonical Wnt signaling at one year of age can re-activate Shh signaling. Expression of Shh itself was not up-regulated, and not much change in the level of Krt19 was detected. However, the expression levels of Shh targets Bra, Sox9, and Col1a1, assayed by immunohistochemistry and qPCR, were up-regulated (Fig. 7). Increase in the mRNA level of Axin2 was also observed, demonstrating activation of canonical signaling pathway. Interestingly, we also observed increased expression of Ccnd1 (shown in Fig. 7), suggesting that the adult NP cells have a potential to enter the cell cycle following activation of Wnt and Shh signaling.

## Discussion

**Figure 8:**
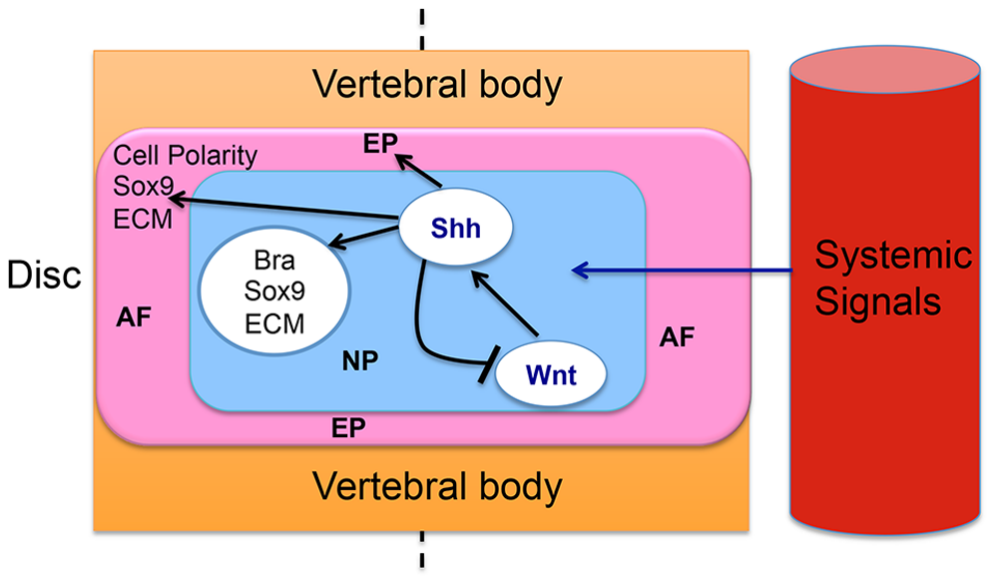
Proposed model: Canonical Wnt signaling activates Shh signaling, which in turn promotes growth and differentiation of all the IVDs by regulating the downstream signaling targets. Shh signaling in turn represses canonical Wnt signaling. These events likely occur at each level of the vertebrae (shown by dotted line), and might be regulated by circulating or systemic signals (Adapted from *Dahia et al., 2012*).

In previous work, we showed that Shh signaling from the NP cells is both necessary and sufficient for many aspects of disc growth and differentiation. This raised the obvious question of what controls Shh signaling in the IVD. One clue to this was the dramatic rise in canonical Wnt signaling caused by Shh signaling inhibition, suggesting that Hh signaling feeds back on the level of Wnt signaling in the NP cells. The current study addressed the role of Wnt signaling during postnatal development of the IVDs and its relationship with the Shh signaling pathway. Here we demonstrated, both in organ culture *in vitro*, and by gene targeting in vivo, that canonical Wnt signaling controls the expression of Shh targets. These two observations; the repression of Wnt signaling by Shh [8], and the control of Shh targets by Wnt signaling (reported here) suggest a feedback loop between Wnt and Shh signaling in the NP (Fig. 8).

In the *in vitro* experiments, doses of BIO and XAV necessary to affect reporters of canonical Wnt signaling were higher than have been previously reported in monolayer cultures [16], [17]. This is probably due to the large size, relative to a cell monolayer, of the organ cultures, and the requirement for the small molecule inhibitors to diffuse into the center of the IVD. However, we feel that the high doses are justified, since they caused the predicted changes in mRNA and protein expression. In particular, the up-regulation of Wnt target genes such as AXIN2 show that these doses are not acting by non-specific cell damage.

We have not explored the mechanism by which each signaling pathway controls the other. Wnt signaling could control Shh signaling either directly by controlling expression of an essential Shh target, or inhibiting the action of a Shh signaling intermediate, or indirectly by controlling functional levels of Shh ligand. For example, if Wnt signaling acts directly on the concentration of cell surface or extracellular matrix proteoglycans, this would affect the local concentrations of Shh ligand. These possibilities will be explored in a separate study. It is possible that Wnt signaling has other effects in the IVD, in addition to its action of Shh signaling.

A feedback loop between Wnt and Shh signaling in the IVD is consistent with a number of recent studies showing interaction between the two pathways during development of the teeth [18], [19], alar plate [20], taste papillae [21], thalamus [22], synovial joints [23] and neural progenitors [24]. Some of these studies have specifically shown that while Wnt upregulates Shh, Shh in turn inhibits Wnt signaling [18], [19], [21]. In addition, Wnt and Shh signaling pathways have been shown to work in parallel to control cell cycle progression, by up-regulating cyclins and cyclin-dependent kinases (cdks) that are critical for cell-cycle progression [24]. It is interesting to note in the case of the IVD, that only the NP cells express Wnts and Shh, but all components of the IVD can respond to them, as shown by previous reporter studies [8] and this work. This suggests that the interactions between these signaling pathways occur in the NP, but the resulting signals control all components of the IVD.

Previous studies [7], [25], [26] have shown that NP cells make a large number of signaling ligands and receptors. It is likely, therefore, that the Wnt/Shh interaction in the NP is only a small part of a larger signal regulatory network. Many recent studies have identified active signaling pathways in the IVD [7], [25]–[33]. It will be important to identify the hierarchy of these pathways. We have previously shown that Shh signaling negatively regulates BMP signaling, and positively regulates TGFβ signaling in the IVD [8], suggesting that Wnt signaling may in turn be an upstream regulator of a number of pathways. More work is required to establish the hierarchy of signaling controls in the disc during its growth and differentiation.

The data presented here place canonical Wnt signaling as an activator of NP cell proliferation and differentiation. Whether Wnts are activators or inhibitors of this process has been controversial [28], [29], [34]–[37]. The effects of Wnt signaling on growth and differentiation are complex. In many tissues tested, canonical Wnt signaling acts to maintain cell proliferation by regulating the expression of cell cycle progression genes such as cyclin D1 [38], c-myc [38]–[40], cyclin D2 [41] and Pitx2 [38]. In addition, cell hyperplasia, in the intestine for example [42], is a consequence of pathological over-activation of Wnt signaling, and as a result Wnt signaling pathway components are therapeutic targets for cancer treatment (reviewed by [43], [44]).

In other studies, however, activation of Wnt signaling has had opposite effects. The addition of LiCl to12-week old rat NP cells in culture caused loss of cell proliferation, and reduced expression of *c-myc* and *cyclin D1* [28]. Similarly, in vascular smooth muscle cells, LiCl caused inhibition of cell proliferation and migration [45]. There are a number of variables to consider here. First the methods used in these different studies. Here we used BIO to inhibit GSK3β and thus increase β-catenin concentration, whereas the studies cited above used LiCl. Although these reagents both inhibit GSK3β, they both have different off-target effects. BIO also inhibits CDK's, to which it is closely related [46]. LiCl does not inhibit CDK's, but inhibits other kinases distinct from those affected by BIO [47], [48], as well as non-kinase targets such as inositol monophosphate and histone deacetylase ([49], [50], reviewed in [51]). These different targets can affect the conclusions. For example, the effect of LiCl on vascular smooth muscle cells was found to be mediated by its action on the transcription factor PGC1α, rather than on Wnt signaling [45]. For these reasons, it is important to show that inhibitors of canonical Wnt signaling (and here we used XAV939) have the opposite effect to the activator chosen. Best of all is to show that activation and inhibition in vivo have opposite effects. Currently this is difficult to achieve for the postnatal NP cells, due to the lack of specific Cre-driver lines. We show here that targeted mutations in the Wntless gene in vivo do cause the same effects as the Wnt inhibitor XAV939 in culture, and opposite effects to those caused by the activator BIO in culture. Even here though, the methods used are not formally identical, for we had to take Wls^−/−^ IVDs from late pre-natal stages (due to postnatal lethality of this mutation), whereas the organ culture experiments were carried out during early postnatal life. Second, differences in experimental design may affect the outcome. In some studies, NP cells cultured in monolayer on plastic dishes were used [28]. It is likely that the cell environment will be different in these experiments due to the presence of signals in the serum used, differences in extracellular matrix molecules, and differences in oxygen tension [52], from the environment of NP cells in the center of IVDs in organ culture in minimal medium. It will be important to standardize experimental conditions before the role of canonical Wnt signaling is finally elucidated. Third, there may be age- and species-differences in response to Wnt agonists and antagonists.

This study also addresses the relationship between cessation of growth and changes in the Wnt and Shh pathways. The NP cells change both their morphology and gene expression patterns as they transition from the rapid growth phase of the spine during the first eight-nine postnatal weeks [2], [7] to a post-growth state. This transition includes the compaction of the NP cells to form a disc-shaped dense mass in the middle of the disc space, surrounded by a proteoglycan-rich matrix. This morphology persists until visible age-related changes in the disc can be seen, by two years of age. By this time, there are few compacted NP cells left in the IVD, and the disc has become filled with loose chondrocytes separated by cartilage matrix. It is not clear whether the chondrocytes present in old discs arise from NP cells, or from the surrounding AF and EP. Although lineage tracing has shown that postnatal NP cells are derived from the embryonic notochord [1], this study did not include the analysis of the cartilage-like cells that invade the IVD late in life, and TUNEL staining suggests that the NP cells slowly die after a brief period of postnatal proliferation [2]. Further studies will be necessary to resolve this point. Also not clear at this time is whether the compacted NP cells represent the chondrocyte-like cells seen in other species, or whether these are equivalent to the more obviously chondrocytes-like cells seen in the old discs of the mouse. The transition from expanded to compacted state of the NP cells after the postnatal growth period is accompanied by a reduction in both Wnt and Shh signaling in the NP, as well as a reduction of expression of many of the characteristic markers of the NP cells. This correlation between reduction of signaling and markers prompted us to test whether the NP cells could be re-activated by stimulating the normal in vivo pathways. There have been many approaches used to stimulate IVD regeneration, including cell transplantation (reviewed by [53] and [54]), and treatment with growth factors [55]–[59]. However, the use of the endogenous signals that control normal growth and differentiation of the disc may be a highly promising approach. The data presented here show that both the compaction of the NP cells, and the reduction in expression of Shh targets in the NP can be at least partly reversed by treatment with either Shh or by activation of canonical Wnt signaling in the disc. The fact that Shh targets can be activated by Wnt signaling suggests that the endogenous pathway of disc growth and differentiation has been at least partly re-activated. It remains to be determined whether all signaling pathways active in the growing disc can be re-activated in this manner, and to what extent this occurs. However, the data do show that re-activation of earlier pathways is possible, and may lead to utilization of developmental pathways as therapies for diseased or aged discs.

In conclusion, we show here that Shh signaling from the NP cells, previously shown to control the expression of cell differentiation and proliferation in the IVD during rapid postnatal growth, is controlled in turn by canonical Wnt signaling, and that feedback between the two pathways controls the level of Wnt signaling. We also show that the activities of both pathways declines in aged discs, concomitantly with age-related morphological changes in the IVD. These changes can be at least partially reversed in organ culture by re-activating either Wnt or Shh signaling. The degree to which age-related changes can occur, and the mechanisms by which Wnt and Shh signaling control each other, and are controlled by circulating (systemic) signals such as hormones to coordinate IVD growth and differentiation in the whole spine, now become important experimental problems (Fig. 8).

## Materials and Methods

### Mice

FVB mice were maintained in accordance with the National Institutes of Health Guide for the Care and Use of Laboratory Animals, and all experiments were carried out in strict accordance with institutional guidelines under Institutional Animal Care and Use Committee (IACUC) approval at Cincinnati Children's Hospital Research Foundation (CCHRF Animal Use Protocol number 2B01001). All studies were carried out using at least three IVDs in each group and repeated three times.

### Generation of *Shh-Cre Wls^flx/flx^* conditional knockout mice

Wls^flx/flx^ conditional knockout mice [60] were interbred with Shh-Cre [61] mouse lines to obtain Shh-Cre; Wls^flx/flx^ mice and genotyped as previously described [14]. Embryos were collected at E18.5 and the lumbar spines were dissected. The IVDs between L1/L2 (L =  lumbar) and L2/L3 IVDs were dissected and stored in OCT molds for histological analysis. The NP cells were collected under a dissecting microscope from IVDs between L3 to L6 and stored in RNAlater (Life Technologies; AM7020) for subsequent qRT-PCR (qPCR) analysis.

### Organ culture of IVDs

Lumbar spines were dissected from postnatal day-four (P4), or one-year-old (1yr) mice, and cultured as described previously [8]. Briefly, immediately after dissection the spines were placed in ice-cold 1x phosphate buffered saline (PBS) containing antibiotics (pen/strep, Sigma; P4333). The IVDs along with the adjacent vertebral growth plates were dissected free from the spines using a stereomicroscope. For the culture experiment, the IVDs were placed on type-IV Collagen (BD Biosciences; 354233) coated Millicel-CM cell culture inserts (Millipore; PICM01250) and cultured in 24-well culture plates in DMEM Ham F-12 containing 1% pen/strep, 1% Nystatin (Sigma; N1638) and supplemented with 1x Insulin-Transferrin-Sodium Selenite supplement (Roche Diagnostics; 11074547001). The culture was maintained at 37°C and 5% CO_2_. Small molecule regulators of Wnt signaling pathway, Stemolecule BIO (Stemgent; 04-003), a Wnt signaling activator [9], and XAV939 (Selleck Chemicals; 284028-89-3), a Wnt signaling inhibitor [11] were used in the study. First, the dose of each reagent was evaluated by carrying a dose- response study (data not shown) to select doses for subsequent studies. The treatment was carried out for either three or five days (t^3^ - t^5^). Vehicle-treated discs served as controls. At the end of the culture period the IVDs were washed three times in cold 1x PBS and either snap frozen in OCT molds for histological evaluation, or the NP cells were dissected and collected in RNAlater (Life Technologies; AM7020) and stored at −80°C for qPCR analysis.

### Histology

Cryosections from cultured IVDs, and from the Wls mutant mice were collected at a thickness of 6 µm in the coronal plane, using a Leica cryostat. Sections were processed immediately, or stored at −20°C for later use. Histology of the vertebrae was analyzed by fixing sections in 2% paraformaldehyde (PFA) and staining with hematoxylin and eosin (H&E). Sections were photographed using a Zeiss axiovert microscope.

### Immunolocalization analysis

Immunoflourescence analysis was carried out on 6 µm-thick mid-coronal cryosections of IVDs from the in vitro and in vivo experiments. The sections were fixed in 2% PFA for 2 minutes, permeabilized using 1x PBS containing 0.25% Triton-x100 for 20 minutes, blocked in blocking buffer for one hour as previously described [2]. The sections were then treated with diluted primary antibody (AXIN2, Abcam, ab32197; Chondroitin sulfate, ab11570, Abcam; Aggrecan, ab3778, Abcam; SHH, S4944, Sigma-Aldrich; GLI1, AF3455, R&D; Brachyury, R&D, AF2085; SOX9, Ab3697, Abcam; Collagen I, GTX41285, Gene Tex, Inc;) overnight at 4°C in a humidified chamber. Next day the sections were washed three times in 1x PBS and treated with 15 µg/mL of the appropriate Cy5-conjugated secondary antibody (Goat anti Mouse; Donkey anti Goat; Goat anti Rabbit; Goat anti Rat, from Jackson ImmunoResearch Laboratories, INC) in blocking buffer for an hour at room temperature and in the dark. The sections were next washed in 1x PBS (twice) and counterstained with POPO3-iodide (1∶5000, Molecular Probes) to stain the cell nuclei, washed in 1x PBS, and mounted in 50% glycerol in 1x PBS and containing 25 µg/mL of the anti-quenching agent DABCO (D27802, Sigma). Images were captured using a Zeiss LSM 510 confocal microscope. Control sections were incubated in the appropriate secondary antibody only, and were all negative (data not shown). The quantification of the immunofluorescence studies three serial sections from each sample were imaged, and the mean pixel density was measured by measuring the fluorescence of the entire disc (when the entire disc is in the field of view), or the NP cells (for data were only the NP cells were in the field of view). The mean pixel density was calculated from the three images. There were at least two replicates from each experiment, and the experiments were repeated three times. The histograms represent data from the replicates of one experiment.

### BrdU incorporation assay

Bromo-deoxy-Uridine (BrdU) assays were carried out using Amersham Cell Proliferation Kit (RPN20; GE Healthcare) as per manufacturer's instructions. BrdU (1∶1000) was added to the culture medium 24 hours before the end of the culture period. The cultured IVDs were washed three times in 1x PBS and snap frozen in OCT molds and cryosections were collected at 6 µm thickness, fixed in 2% PFA and washed thrice in 1x PBS. For the BrdU staining, the sections were pretreated with 2% H_2_0_2_ diluted in methanol to quench the endogenous peroxidase activity followed by three washes in 1x PBS. The sections were next incubated with anti-BrdU antibody provided with the kit for 2 hours at room temperature, followed by 30 minutes incubation with HRP-conjugated secondary antibody. To visualize the BrdU positive cells under the microscope, the sections were treated with the Metal enhanced DAB Substrate kit (34065; Thermo Scientific), and the nuclei were counter-stained pink with nuclear fast red (N3020; Sigma). The sections were dehydrated and mounted in xylene based mounting medium. Sections were photographed using a Zeiss axiovert microscope and BrdU-positive NP cells from the total NP cells on individual section of different treatment groups were counted using the Axiovision software.

### RNA extraction and Real-time qPCR analysis

Changes in gene expression in the NP cells were determined by qPCR analysis as per the MIQE guidelines [62]. RNA was isolated from the NP cells stored in RNAlater using RNeasy micro kit (QIAGEN; 74004). Reverse transcription was carried out using SupperScript III First Strand Synthesis system (Invitrogen; 18080-051). Taqman probes (Life Technologies) and TaqMan fast universal PCR master mix (Applied Biosystems; 4352042) were used to determine the levels of gene expression using StepOnePlus Real-Time PCR system (Life Technologies; 4376598) (details on TaqMan probes: *Gli1*- Mm00494654_m1; *Ptch1*- Mm00436026_m1; *Shh*- Mm00436528_m1; *Wnt4*- Mm01194003_m1; *Wnt5a*- Mm00437347_m1; *Wnt11*- Mm00437328_m1; *Fzd7*- Mm00433409_s1; *Fzd10*- Mm00558396_s1; *Wls*-Mm00509695_m1; *Lef1*-Mm00550265_m1; *Bra*- Mm01318252_m1; *Sox9*- Mm00448840_m1; *Krt19*- Mm00492980_m1; *Col2a1*- Mm01148576_m1; *Acan*- Mm00545794_m1; Col1a1-Mm00801666_g1; Ccnd1-Mm00432359_m1). Two reference genes 18S and GAPDH were used for normalization (18S-Mm03928990_g1; GAPDH-Mm99999915_g1).

### Statistical analysis

The means and s.d. of the values obtained from individual experiments were calculated and represented as charts. Each experiment was repeated three times with two to three biological replicates in each group. Significance was calculated using unpaired T-test or one-way ANOVA.

## Supporting Information

Figure S1 **Shows expression of CHSO4 protein expression (red) in P4t0 lumbar vertebrae growth plate (LV-GP).** The expression of CHSO4 can be seen was much higher in the disc space. The nuclei are counter stained blue with DAPI. Scale bar  =  50 µm.
(TIF)
