# Supplementary figures and images for: Wnt Signaling Activates Shh Signaling in Early Postnatal Intervertebral Discs, and Re-Activates Shh Signaling in Old Discs in the Mouse
Source: PLoS One. 2014 Jun 3;9(6):e98444. doi: 10.1371/journal.pone.0098444 (PMC4043533; doi:10.1371/journal.pone.0098444)

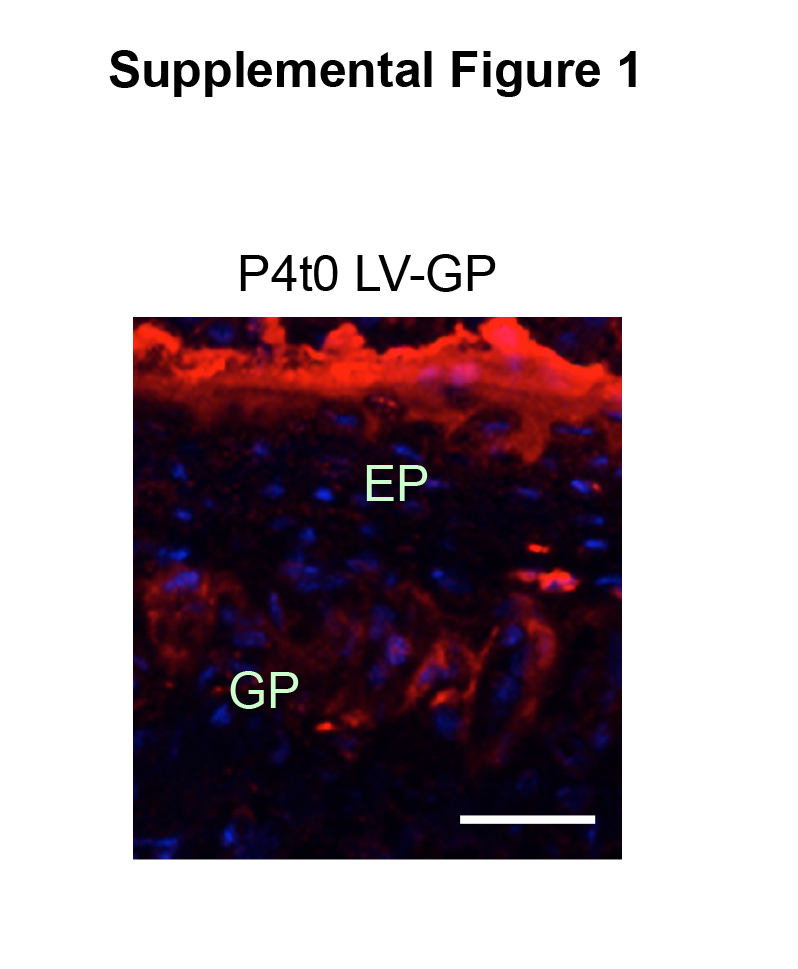

Supplement: Figure S1 — Shows expression of CHSO4 protein expression (red) in P4t0 lumbar vertebrae growth plate (LV-GP). The expression of CHSO4 can be seen was much higher in the disc space. The nuclei are counter stained blue with DAPI. Scale bar = 50 µm. (TIF) [file pone.0098444.s001.tif]
